# Associations between patients’ risk attitude and their adherence to statin treatment – *a population based questionnaire and register study*

**DOI:** 10.1186/s12875-016-0423-1

**Published:** 2016-03-09

**Authors:** Benedicte Lind Barfoed, Maja Skov Paulsen, Palle Mark Christensen, Peder Andreas Halvorsen, Trine Kjær, Mogens Lytken Larsen, Pia Veldt Larsen, Jesper Bo Nielsen, Jens Søndergaard, Dorte Ejg Jarbøl

**Affiliations:** Research Unit of General practice, Department of Public Health, University of Southern Denmark, JB Winsløws Vej 9A, 5000 Odense C, Denmark; Danish Quality Unit of General Practice, JB Winsløws Vej 9A, 5000 Odense C, Denmark; General Practitioners Lærkevej, Lærkevej 14, 5450 Otterup, Denmark; Department of Community Medicine, University of Tromsø, The Arctic University of Norway, 9037 Tromsø, Norway; Centre of Health Economics Research, COHERE, University of Southern Denmark, Campusvej 55, 5230 Odense M, Denmark; Department of Cardiology, Aalborg University Hospital, Sdr. Skovvej 15, 9000 Aalborg, Denmark; Center for Clinical Epidemiology, Odense University Hospital, Sdr Boulevard 29, 5000 Odense C, Denmark; Research Unit of Clinical Epidemiology, Institute of Clinical Research, University of Southern Denmark, Sdr. Boulevard 29, 5000 Odense C, Denmark

**Keywords:** Cardiovascular, Statins, Risk attitude, Adherence, General practice

## Abstract

**Background:**

Poor adherence to medical treatment may have considerable consequences for the patients’ health and for healthcare costs to society. The need to understand the determinants for poor adherence has motivated several studies on socio-demographics and comorbidity. Few studies focus on the association between risk attitude and adherence. The aim of the present study was to estimate associations between patients’ adherence to statin treatment and different dimensions of risk attitude, and to identify subgroups of patients with poor adherence.

**Methods:**

Population-based questionnaire and register-based study on a sample of 6393 persons of the general. Danish population aged 20–79. Data on risk attitude were based on 4 items uncovering health-related as well as financial dimensions of risk attitude. They were collected through a web-based questionnaire and combined with register data on redeemed statin prescriptions, sociodemographics and comorbidity. Adherence was estimated by proportion of days covered using a cut-off point at 80 %.

**Results:**

For the dimension of health-related risk attitude, “Preference for GP visit when having symptoms”, risk-neutral and risk-seeking patients had poorer adherence than the risk-averse patients, OR 0.80 (95 %-CI 0.68–0.95) and OR 0.83 (95 %-CI 0.71–0.98), respectively. No significant association was found between adherence and financial risk attitude. Further, patients in the youngest age group and patients with no CVD were less adherent to statin treatment.

**Conclusion:**

We find some indication that risk attitude is associated with adherence to statin treatment, and that risk-neutral and risk-seeking patients may have poorer adherence than risk-averse patients. This is important for clinicians to consider when discussing optimal treatment decisions with their patients. The identified subgroups with the poorest adherence may deserve special attention from their GP regarding statin treatment.

## Background

Poor adherence to medical treatment may have considerable consequences for patients’ health and for healthcare costs to society [1]. Adherence is a measure of the degree to which the patient follows the treatment as agreed with the doctor [2]. It is estimated that adherence to long-term drug treatment of chronic disease is about 50 % in developed countries [3]. Adherence is particularly low for preventive therapy and for treatment of diseases that do not cause symptoms perceivable to the patient [4, 5]. It is well documented that patients’ comorbidity, co-medication, and socioeconomic status are important determinants of poor adherence [6–8]. Furthermore qualitative research on adherence has focused on how persons’ everyday conditions such as family life, holidays, and leisure schedules and societal eating rules influence on the management of adherence and adaptation of medical treatment [9, 10]. During the past decades the influence of risk attitude on health behaviour and health outcome has received little but an increasing attention in the literature [11–15]. Risk attitude is highly individual and can be influenced by fear, sense of control, personal experiences, socioeconomic conditions and the perceived severity of a condition [16–19]. This study is one of the first to examine associations between risk attitude and adherence to statin treatment and to examine whether risk attitude can explain some of the previous observed person-specific heterogeneity by explicitly incorporating the effect of risk attitude into our analysis of adherence to statin treatment. The aim of the study was to estimate associations between adherence and different dimensions of risk attitude, and to identify subgroups with low adherence.

## Methods

### Sources of data

**Fig. 1:**
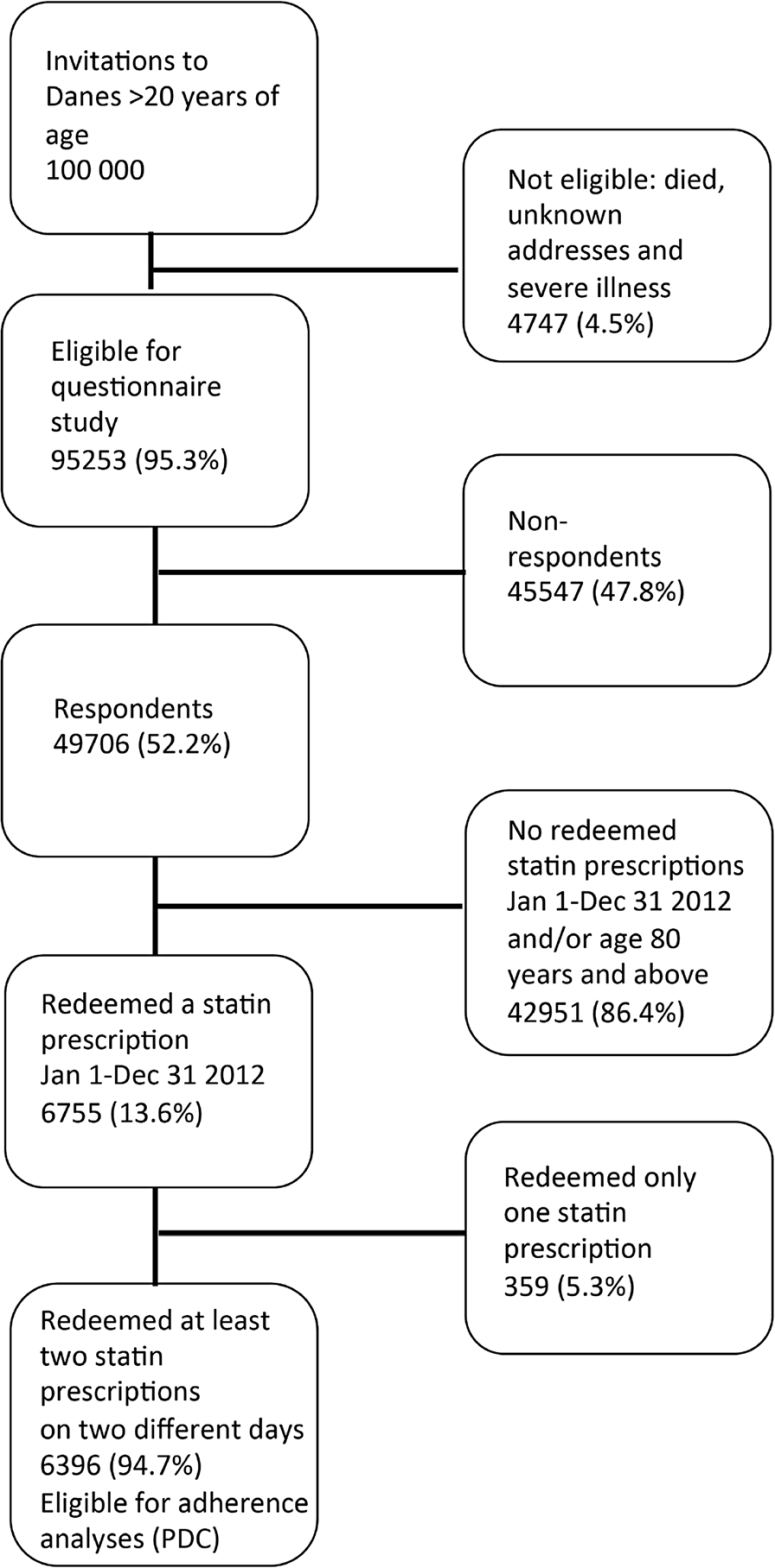
Flowchart of patient sampling

Data on risk attitude were obtained through a large questionnaire study, The Danish Symptom Cohort, carried out from June to December 2012 with the overall aim of gaining knowledge of symptoms, healthcare-seeking and related personality characteristics in the adult Danish population. Prior to sending out the questionnaire, it was pilot tested in its entirety for content validity, relevance, acceptability and feasibility. The data quality, response rate, floor and ceiling effects, score ranges, means and standard deviations of single items and scores were assessed by a field test from the adult Danish population. Details about the development of the questionnaire are reported elsewhere [20]. The study was designed as a nationwide cohort study of 100,000 persons aged 20 years or above from the general Danish population with baseline data collected in a web-based survey (Fig. 1). The median age of the respondents was 52 years (interquartile range (IQR) 40–64) compared to 50 years (IQR) 36–66) for non-respondents. Some 53.2 % of the respondents were women compared to 48.6 of the non-respondents [21].

Eligible participants for the present study were between 20–79 years of age and had redeemed at least 2 prescriptions for statin treatment between 1 January 2012 and 31 December 2012.

### Risk attitude

Risk attitude has previously been measured using case vignettes and statements representing various attitudes for the respondents to agree or disagree with [14, 18]. There is some indication that risk attitude might be domain specific [19]. To allow for the measurement of risk attitude in a broader perspective, we therefore chose to elicit risk attitude in two domains, financial and health-related. Financial risk attitude was measured by a lottery choice experiment (Table 1), which has previously been shown to predict health behaviours such as alcohol consumption, smoking, seat belt use [11] and patients’ treatment choice [22]. In order to elicit different dimensions of health-related risk attitude three items were developed specifically for this study, see Table 1.

**Table 1:** Risk attitude items and response categories

Item wording	Risk-averse		Risk-neutral	Risk-seeking	
Imagine that you unexpectedly inherited DKK 10,000 (approximately USD 2,000) from a distant relative. Subsequently you have the possibility of participating in a lottery with an equal chance of doubling the money or losing the money. That means that there is a 50 % chance of you winning DKK 20,000 and a 50 % chance of losing the DKK 10,000.	*I choose not to participate in the lottery*		*I do not know*	*I choose to participate in the lottery*	
What do you choose? *(abbreviated: “Financial”)*					
I focus a lot on having a healthy behaviour and prefer to avoid risks that can affect my health. *(abbreviated: “Focus on healthy behaviour”)*	*Completely agree*	*Tend to agree*	*Yes and no*	*Tend to disagree*	*Completely disagree*
When I experience symptoms, I generally count on them passing. *(abbreviated: “Count on symptoms passing”)*	*Completely disagree*	*Tend to disagree*	*Yes and no*	*Tend to agree*	*Completely agree*
I do not like to take chances regarding my health and prefer to see my GP once too often than once too late. *(abbreviated: “Preference for GP contact regarding my health”)*	*Completely agree*	*Tend to agree*	*Yes and no*	*Tend to disagree*	*Completely disagree*

### Register data

The questionnaire data were combined with register data from the Danish National Prescription Register (DNPR), the National Patient Register (NPR), and demographic databases from Statistics Denmark. DNPR contains data on all redeemed prescriptions in Denmark since 1994 [23]. From the DNPR we included the following information for each redeemed prescription: Identification of the dispensed product using the Anatomical Therapeutic Chemical Classification System (ATC), number of packages and pack size dispensed, patients’ personal registration number and date of prescription redemption. In order to estimate adherence and duration of treatment we included data on redeemed prescriptions for statin treatment (ATC C10AA) from 1 January 1996 to 31 December 2012. As markers of cardiovascular disease (CVD), we included data on redeemed prescriptions for platelet aggregation inhibitors (ATC B01AC04, −22 & -24) from 1 January 1996 to 31 December 2012. NPR contains complete individual-level data from Danish hospitals on all admissions since 1977 [24]; including date, hospital, type of contact and diagnoses. From NPR we included data on in-patient episodes of CVD from 1 January 1996 to 31 December 2013. From demographic databases from Statistics Denmark we included data on highest attained educational level, income, cohabitation status and labour market status [25–27] in 2012.

### Adherence

Adherence was measured as the proportion of days covered (PDC) [28], measuring the number of daily doses of medication a patient has purchased relative to the length of a defined study period. Since statin tablets exist in all clinically relevant doses e.g. 10, 20, 40 and 80 mg, we find it reasonable to assume that one tablet a day equals one daily dose [29], rather than patients taking e.g. two tablets or half a tablet a day. The study period was defined as the time from index-date to end-date. For each patient the index-date was the date of the first redeemed prescription for a statin in 2012, and the end-date was the date of the last redeemed prescription in the year following the index date. This means that the end-date could be any date in 2013 depending on the index-date for the individual patient. A patient’s PDC was calculated by dividing the total number of tablets from all redeemed prescriptions during the study period, excluding the redeemed prescription on the end-date, into the number of days in the study period. Patients with a PDC above 0.8 were categorised as adherent and patients with a PDC equal to or below 0.8 as non-adherent [30].

### Comorbidity

As comorbidity may be related to both risk attitude and adherence it was considered a potential confounder. We measured comorbidity in two different ways: General comorbidity using the Charlson Comorbidity Index [31] and using CVD. The effect of CVD might be different from general comorbidity, since patients with CVD may be more likely to take statins than patients with other comorbidities [32]. We therefore treated both Charlson Comorbidity index and CVD as confounders in the analyses as proposed by Benner et al. [32].

The NPR was used to calculate the Charlson Comorbidity Index for each patient and to identify patients with CVD. Patients were categorised as having CVD, if they had 1) been admitted with diagnoses of stroke, acute coronary syndrome and/or complications and angina or had undergone coronary bypass graft or percutaneous coronary intervention or 2) had redeemed a prescription for clopidogrel, prasugrel and ticagrelor, platelet aggregation inhibitors used as secondary prevention to prevent new myocardiac infarction.

### Statistical methods

For each of the 4 risk attitude items, responses were categorised into 3 groups: “Risk-seeking”, “risk-averse” and “risk-neutral” (Table 1) according to the terminology used in standard economic theory [33]. Logistic regression was used to estimate associations between adherence and each of the 4 risk attitude dimensions, as well as age group (20–39 years, 40–59 years, 60–79 years), gender, highest attained educational level (<10 years, 10–12 years, >12 years), income (1st quartile, 2nd + 3rd quartile, 4th quartile), cohabitation (single, married/cohabiting) labour market status (working, retirement pension, not in the workforce), duration of treatment (<1 year, 1–2 years, 2–5 years, 5–10 years, >10 years), Charlson Comorbidity Index and CVD [34, 35].

The analyses were adjusted for age, gender, highest attained educational level and cohabitation, the Charlson Comorbidity Index and CVD. Missing values were considered missing at random. STATA release 13.0 (StatCorp, College Station, TX) was used for all statistical analyses.

## Results

Of the 49,706 initial respondents to the Danish Symptom Cohort, some 6755 patients met the inclusion criteria for the present study. Of the 6755 patients 359 redeemed only one statin prescription in 2012, leaving 6396 patients eligible for adherence analyses (Fig. 1 & Table 2).

**Table 2:** Distribution of risk attitude, socioeconomic factors, duration of treatment and comorbidity, *n* = 6396*

	N (%)
Risk attitude, Financial	
Risk-averse	5188 (81.1)
Risk-neutral	512 (8.0)
Risk-seeking	696 (10.9)
Risk attitude, health: Focus on healthy behaviour	
Risk-averse	4153 (64.9)
Risk-neutral	1436 (22.5)
Risk-seeking	807 (12.6)
Risk attitude, health: Count on symptoms passing	
Risk-averse	776 (12.1)
Risk-neutral	591 (9.2)
Risk-seeking	5029 (78.6)
Risk attitude, health: Preference for GP contact regarding my health	
Risk-averse	3572 (55.9)
Risk-neutral	1244 (19.5)
Risk-seeking	1580 (24.7)
Gender	
Male	3544 (55.4)
Female	2852 (44.5)
Age (years)	
20–39	70 (1.1)
40–59	1781 (27.9)
60–79	4545 (71.1)
Income	
Low (1st quartile)	957 (15.0)
Medium (2nd + 3rd quartile)	3504 (54.8)
High (4th quartile)	1933 (30.2)
Highest attained educational level (years)	
< 10	1757 (27.9)
10–12	2992 (47.5)
> 12	1551 (24.6)
Cohabitation status	
Single	1399 (21.9)
Married/cohabiting	4995 (78.1)
Labour market status	
Working	2574 (40.2)
Retirement pension	3193 (49.9)
Not in the workforce	629 (9.8)
Duration of statin treatment	
< 1 year	539 (8.4)
1–2 years	547 (8.6)
2–5 years	3089 (48.3)
5–10 years	1526 (23.9)
< 10 years	695 (10.9)
Comorbidity	
CVD	
No CVD	5340 (83.5)
CVD	1056 (16.5)
Charlson	
None	3593 (55.6)
1	1491 (23.8)
≥ 2	1289 (20.6)

*Up to 1.5 % missings in registers

For the financial risk attitude item, some 5188 patients (81.1 %) were categorised as risk-averse. For the risk attitude items “Focus on healthy behaviour”, “Count on symptoms passing” and “Preference for GP contact regarding my health”, 4153 (64.9 %), 776 (12.1 %) and 3572 (55.9 %) patients, respectively, were categorised as risk averse (Table 2).

Overall, some 5280 (82.6 %) of the 6396 respondents were adherent to their medication with statins. For the risk attitude dimension “Preference for GP contact regarding my health”, the risk-neutral and risk-seeking patients had significantly poorer adherence than the risk-averse patients, OR 0.80 (95 %-CI 0.68–0.95) and OR 0.83 (95 %-CI 0.71–0.98), respectively (Table 3). The dimensions of financial risk attitude and the risk attitude dimensions “Focus on healthy behaviour” and “Count on symptoms passing” were not significantly associated with adherence. Patients in the youngest age group had poorer adherence than older patients OR 2.61 (95 % CI 1.52–4.47), and patients in the workforce had poorer adherence than patients on retirement pension OR 1.32 (95 %-CI 1.14–1.52). Respondents with CVD had better adherence to treatment, OR 1.36 (95 %-CI 1.11–1.67) compared to respondents without CVD (Table 4).

**Table 3:** Associations between risk attitude and adherence (PDC > 80 %*), *n* = 6396**

	Proportion of adherent patients (%)	OR crude (95 % CI)	*P*-value	OR adj.*** (95 % CI)	*P*-value
Risk attitude, Financial					
Risk-averse	4291/5188 (82.7)	1		1	
Risk-neutral	421/512 (82.2)	0.97 (0.76–1.23)	0.783	0.93 (0.73–1.19)	0.560
Risk-seeking	568/696 (81.6)	0.93 (0.76–1.14)	0.472	0.94 (0.76–1.16)	0.562
Risk attitude, health:					
Focus on healthy behaviour					
Risk-averse	3464/4153 (83.4)	1		1	
Risk-neutral	1167/1436 (81.3)	0.86 (0.74–1.01)	0.064	0.88 (0.75–1.03)	0.116
Risk-seeking	649/807 (80.4)	0.82 (0.67–0.99)	0.039	0.84 (0.69–1.03)	0.096
Risk attitude, health: Count on symptoms passing					
Risk-averse	644/776 (83.0)	1		1	
Risk-neutral	489/591 (82.7)	0.98 (0.74–1.31)	0.904	0.99 (0.74–1.33)	0.948
Risk-seeking	4147/5029 (82.5)	0.96 (0.79–1.18)	0.718	0.91 (0.74–1.13)	0.395
Risk attitude, health: Preference for GP contact regarding my health					
Risk-averse	2996/3572 (83.8)	1		1	
Risk-neutral	1000/1244 (80.4)	0.79 (0.67–0.93)	0.005	0.80 (0.68–0.95)	0.011
Risk-seeking	1284/1580 (82.6)	0.83 (0.71–0.97)	0.021	0.83 (0.71–0.98)	0.025

*Adherence was defined as a PDC (proportion of days covered) above 80 %

**Up to 1.5 % of the data were missing in registers

***We adjusted for age group, Charlson Comorbidity Index and CVD, highest attained educational level, cohabitation status and duration of statin treatment

**Table 4:** Associations between adherence (PDC > 80 %*) and socioeconomic factors, cohabitation, duration of treatment and comorbidity, respectively *n* = 6396**

	Proportion of adherent patients (%)	OR crude (95 % CI)	*P*-value	OR adj.*** (95 % CI)	*P*-value
Gender					
Male	2903/3544 (81.9)	1		1	
Female	2372/2852 (83.2)	1.08 (0.95–1.23)	0.243	1.13 (0.98–1.29)	0.089
Age (years)					
20–39	47/70 (32.9)	1		1	
40–59	1429/1781 (80.2)	1.99 (1.19–3.32)	0.009	2.20 (1.28–3.78)	0.004
60–79	3804/4545 (83.7)	2.51 (1.51–4.16)	<0.001	2.61 (1.52–4.47)	<0.001
Income					
Low (1st quartile)	807/957 (84.3)	1		1	
Medium (2nd + 3rd quartile)	2916/3504 (83.2)	0.92 (0.75–1.12)	0.414	0.93 (0.76–1.15)	0.508
High (4th quartile)	1555/1933 (80.4)	0.76 (0.62–0.94)	0.011	0.82 (0.65–1.04)	0.098
Highest attained educational level (years)					
< 10	1474/1757 (83.9)	1		1	
10–12	2470/2992 (82.6)	0.91 (0.78–1.06)	0.235	0.93 (0.79–1.10)	0.405
> 12	1256/1551 (81.0)	0.82 (0.68–0.98)	0.028	0.85 (0.71–1.02)	0.088
Cohabitation status					
Single	1157/1399 (82.7)	1		1	
Married/cohabiting	4121/4995 (82.5)	0.99 (0.84–1.15)	0.862	1.01 (0.86–1.19)	0.901
Labour market status****					
Working	2054/2574 (79.8)	1		1	
Retirement pension	2700/3193 (84.6)	1.37 (1.21–1.59)	<0.001	1.32 (1.14–1.52)	<0.001
Not in the workforce	526/629 (83.6)	1.29 (1.03–1.63)	0.030	1.25 (0.99–1.59)	0.064
Duration of statin treatment					
< 1 year	456/539 (84.6)			1	
1–2 years	447/547 (81.7)	0.81 (0.59–1.12)	0.205	0.82 (0.59–1.14)	0.244
2–5 years	2490/3089 (80.6)	0.76 (0.59–0.97)	0.029	0.74 (0.57–0.96)	0.024
5–10 years	1290/1526 (84.5)	0.99 (0.76–1.31)	0.971	0.95 (0.72–1.27)	0.741
> 10 years	597/695 (85.9)	1.12 (0.81–1.52)	0.523	1.02 (0.73–1.41)	0.924
Comorbidity					
CVD					
No CVD	4366/5340 (81.8)	1		1	
CVD	914/1056 (86.6)	1.43 (1.19–1.74)	<0.001	1.36 (1.11–1.67)	0.003
Charlson					
None	2858/3593 (81.8)	1		1	
1	1240/1491 (83.2)	1.10 (0.93–1.29)	0.256	1.00 (0.84–1.18)	0.984
≥ 2	1089/1289 (84.5)	1.21 (1.02–1.44)	0.032	1.07 (0.89–1.28)	0.464

*Adherence was defined as a PDC (proportion of days covered) above 80 %

**Up to 1.5 % of the data were missing in registers

***We adjusted for age group, Charlson Comorbidity Index and CVD, highest attained educational level, cohabitation status and duration of statin treatment

****Labour market status was not adjusted for age group because of the close correlation

Kendall’s tau correlation between CVD and the Charlson Comorbidity Index was low (τ = 0.29). The interactions between risk attitude, CVD and Charlson Comorbidity Index, respectively, and adherence were insignificant (*p*-values 0.607 and 0.827).

## Discussion

### Summary findings

The present study considered associations between adherence and four different dimensions of risk attitude in relation to health and finance. Significant associations were found between the risk attitude for the health dimension “Preference for healthcare-seeking when having symptoms” and adherence to statin treatment, where risk-neutral and risk-seeking patients had poorer adherence than risk-averse patients. No associations were found between adherence and the other dimensions of risk attitude. Patients in the youngest age group and patients without CVD were the least adherent. Patients on retirement pension were more adherent than patients in the workforce.

### Implications for practice and future research

Statins are only beneficial, if patients use them consistently and long-term. If risk attitude is an unchangeable personality characteristic, maybe clinicians should support some risk-seeking patients in not using statins instead of using them with poor adherence. Here interventions to help clinicians communicate adequately with their patients about risks and benefits of medical treatment and involving patients in decisions to prescribe medical treatment are recommended to enhance adherence [8].

Literature addressing the relationship between risk attitude and adherence to medical treatment choice is sparse, but a few studies focus on related topics. Prosser et al. found that the more risk-seeking an individual is, the more likely he or she is to choose no treatment [22], but the study is limited by a small sample size and self-reported adherence. King et al. studied associations between risk-taking attitude and cardiac care in a large cohort study and suggest that patients’ risk attitude may contribute to differences in the use of cardiac procedures [36]. The study of King et al. is relevant to our findings as adherence might be considered as a step on the causal pathway between risk attitude and cardiac care. Our results are in line with Benner et al. finding that patients with CVD were significantly more adherent to statin treatment than patients with no CVD [32]. This may be a result of some cardiovascular conditions causing symptoms which the patients perceive as severe and which might induce them to be more careful with their daily medication intake and thus more adherent as suggested by the WHO [1].

Questionnaires are a common way of measuring personality characteristics such as risk attitude [18, 19, 36, 37], but they might be impractical to use in everyday clinical practice. The present findings could, however, serve as inspiration in developing a tool to be used by GPs in the clinical consultation in order to guide them about likely adherence. Other interesting issues for future research would be to explore associations between risk attitude and primary non-adherence and/or early discontinuation of treatment i.e. patients who decides not to embark on statin treatment while having received a prescription or patients who abandon treatment after having redeemed their first prescription.

The present findings support previous research in underlining that clinicians should consider potential adherence issues when discussing optimal treatment decisions with their patients. Further, the least adherent subgroups may deserve special attention from their GP. Targeting clinicians’ attention to these patient groups may improve adherence on a more general basis. Thus it is possible that the findings regarding adherence might be transferred to other areas such as other asymptomatic conditions, vaccination and screening programmes.

### Strengths and limitations

We chose to study patients with cardiovascular risk and statin treatment since dyslipidemia is a chronic and asymptomatic condition and represents a major modifiable cardiovascular risk factor which can be reduced with statins to decrease overall cardiovascular risk [38]. Further, statins are usually administered as one tablet daily, which makes pharmocoepidemiological analysis more straightforward than for drugs with more complex administrations.

A major strength of the study is the large representative sample from the general Danish population obtained through the Danish Symptom Cohort. Further, in this study we estimated adherence using valid registers of on-going prescription redemption, which is considered a fairly accurate way of estimating actual medication use in large populations [28]. A broad range of measures of adherence using prescription databases exists [39]. The method of PDC has several advantages: It includes a dimension of long-term use in the analysis by defining the proportion of days the patient has tablets available over a long period. It measures the degree of perseverance and consistency in daily medication taking behaviour. By using adherence and PDC with a fixed cut-off point, we elucidate contrasts between those patients who are adherent on a daily basis and those who are not [40].

In the analyses, we adjusted for two types of comorbidity, general comorbidity using the Charlson Comorbidity Index and CVD. However, we were unable to adjust for psychiatric comorbidity such as depression. Consequently, we cannot rule out that this may have confounded our results.

The response rate to the Danish Symptom Cohort questionnaire was 52.2 %, which may raise the question whether risk-averse patients might be more likely to complete the questionnaire thus introducing a source of bias into the results. As in most questionnaire studies, the response-rate could be a limitation. However, risk attitude is considered to be more dependent on age and gender [41], and these characteristics were well balanced between respondents and non-respondents. To elicit the different dimensions of risk attitude, the items were constructed in different ways: Both a case vignette (a lottery), as the financial risk attitude item, and statements representing various health risk attitudes that the respondents were asked to agree or disagree with were used. The application of different item constructs strengthened the basis for estimating risk attitude. For the risk attitude on health “Count on symptoms passing”, the responses “completely agree” or “mostly agree” were categorised as risk-seeking (see Table 1). It may be argued that the wording of this statement might imply a degree of prudence in the way that some extent of patience with emerging symptoms before healthcare seeking may be perceived as wise. The wording of the risk attitude on health “Preference for GP contact regarding my health” could be perceived as leading encouraging people to disagree to take chances and see a GP to late. Yet we see great variation in the responses to that item with 44.2 % answering “yes and no”, “tend to disagree” or “completely disagree” and thereby being categorised as risk-neutral or risk-seeking.

## Conclusion

We found that the risk-seeking, the young and those without CVD were less likely to be adherent to statin therapy. The association between risk attitude and adherence was found for one of the three dimensions of health-related risk attitude, “Preference for GP visit when having symptoms”, but not for risk attitude in the financial domain. To the extent that our findings are confirmed in future studies, health related risk attitude might become an important issue in conversations between patients and their GPs.

## Ethical approval

The project was approved by the Danish Data Protection Agency (jour. no. 2011-41-6651) and the Danish Health and Medicines Authority (6-8011-942/1). The regional scientific ethics committee was notified, but according to Danish law the project needed no approval since it did not imply interventions or biomedical samples from the patients. All respondents to the questionnaire signed a formula giving consent to the use of their answers in a research context and to obtaining data from health registers and medical records exclusively for research purposes.
